# Numerical Simulation of Tailings Flow from Dam Failure over Complex Terrain

**DOI:** 10.3390/ma15062288

**Published:** 2022-03-20

**Authors:** Yi Yang, Xiaowen Zhou, Xiaoyu Chen, Chao Xie

**Affiliations:** 1 School of Civil Engineering and Transportation, South China University of Technology, Guangzhou 510641, China; msyangyi@mail.scut.edu.cn (Y.Y.); xwzhou@scut.edu.cn (X.Z.); xiaoyuchen0317@163.com (X.C.); 2 State Key Laboratory of Subtropical Building Science, South China University of Technology, Guangzhou 510641, China; 3 School of Architecture and Urban Planning, Guangdong University of Technology, Guangzhou 510090, China

**Keywords:** tailings dam failure, Bingham model, CFD, inundation area and depth, protection and control measures

## Abstract

A tailings dam failure can lead to disastrous impacts on people’s livelihood and the surrounding ecological environment. Due to interactions among water, tailings and ground, the mechanism of a tailings flow is more complicated than that of a flood flow. In this paper, the tailings flow is regarded as a homogeneous and incompressible non-Newtonian fluid. Its rheological properties were studied through rheological tests conforming to the Bingham model. The rheological parameters were further used in a Computational Fluid Dynamics (CFD) simulation over complex terrain to explore the tailings flow characteristics. The method was validated with experimental results of a non-Newtonian dam-break flow from literature. The flow characteristics, including flow velocity, runout distance, inundation area and depth, were analyzed in the case of the Dagangding tailings dam. The results showed that the downstream railway and village would not be affected in a conservative scenario. Finally, the effects of two measures for preventing tailings flow hazards were discussed. Setting the check dam and planting grasses and trees can effectively mitigate the damage of tailings flow.

## 1. Introduction

A tailings dam is a permanent infrastructure constructed by intercepting valleys or enclosing lands to form a tailings pond to store discharged tailings from mines after ore separation [1,2]. Once a tailings dam collapses, the downstream region will be seriously affected by the sudden massive release of tailings. Take, for example, the 2019 Brumadinho breach dam event in Brazil. Almost 1.17 × 10^7^ m^3^ tailings were released, covering a distance of about 9 km and an inundation area of approximately 3 × 10^6^ m^2^. The tailings flow killed 259 people, and 11 were reported missing [3]. Hundreds of tailings dam failure cases have been recorded worldwide [4,5,6,7]. The total number of tailings dams in China ranks first in the world. Over forty accidents occurred from 2005, mainly caused by dam-break [8,9,10]. Therefore, it is crucial to investigate the characteristics of tailings flow from dam failure for safe production and environmental protection.

The mechanical law of tailings flow is quite complicated, involving multiple interdisciplinary fields, including fluid mechanics, sediment transport and geological hazards [9,11,12]. Although neither systematic theories nor specific guidelines of tailings flow have been established, current research has progressed on the methods of empirical formula, model test and numerical simulation. The empirical formula approach estimates the runout distance by analyzing the relationships among dam parameters, tailings characteristics and topographic features [13,14,15]. It is a relatively rough and straightforward technique based on plenty of statistical data. Compared to the empirical formula approach, the model test method has unique advantages on a specific project, where accurate dynamics profiles can be portrayed [16,17,18,19,20]. It has the limitation of reflecting the practical behaviour of tailings flow due to the difficulty in determining the mapping between a field- and model-length scale [21]. Thus, it remains an open issue for the application of these two methods.

The numerical simulation technique appears as an effective tool to investigate dam-break-type flow, resulting from its capability in realistic model characterisation. [22,23,24,25,26,27,28,29]. Two typical numerical methods are widely adopted on the simulation of fluid dynamics, namely, Smoothed Particle Hydrodynamics (SPH) and Computational Fluid Dynamics (CFD). The literature has reported the constitutive model development for either SPH or CFD. For example, Pastor et al. [24] proposed a depth-integrated model to simulate flows caused by the failure of tailings dams. The model is enhanced and applied by Dutto et al. [25] on the simulation of different types of flowslides through the SPH method. Similarly, Babaoglu and Simms [26] also adopted the SPH method, incorporating a ‘bi-viscosity’ model to simulate the non-Newtonian behaviour. Wang et al. [27,28] extended the method to predict the runout routing from two-dimensional to three-dimensional situations. Even though SPH has popularity in dam-break-flow problems, it has limitations in the accurate description of the free surface, which can cause significant numerical error. In contrast, the CFD method has the advantage of tracking surface locations, especially for challenging boundary conditions. Marsooli and Wu [29] set up a three-dimensional CFD model to simulate dam-break flow, and results indicate the suitability of the CFD method for handling the complicated boundary condition of uneven beds. Both Pirulli et al. [30] and Yu et al. [31] implemented a numerical framework to simulate the motion of rapid flow movements, aiming at practical cases. Nonetheless, fluid rheological properties determination that limits the application of the CFD method in simulating tailings flow remains a key issue.

This study investigated the calibration of the rheological properties from the experiment. Then, the obtained rheological values served as the input parameters in numerical simulation. The tailings flow characteristics, including flow velocity, runout distance, inundation area and depth, were explored, focusing on the 3D complex terrain within a commercial CFD framework (ANSYS-CFX). Furthermore, the effects of measures to prevent tailings flow hazards were discussed.

The main contributions of this study are threefold. (1) The rheological properties of tailings slurry versus the different mass concentrations were investigated. It was found that this kind of slurry, which is composed of lead-zine mine tailings with about two-thirds fine particles (0.075~0.25 mm), conforms to the Bingham model. Furthermore, the results demonstrated a non-linear relationship between the mass concentration and yield shear stress or dynamic viscosity. (2) This study establishes a framework linking the modelling of realistic geological topology features to the description of flow characteristics. The principal components of features, such as sharp turning, varying roughness, and sudden flattening, can be accurately captured by this framework. Correspondingly, the flow characteristics composed of flow velocity, runout distance, inundation area and depth can be elaborated. (3) The effects of measures to preventing tailings flow hazards were investigated. Both setting a check dam and planting trees and grasses can mitigate the damage of tailings flow.

## 2. Methods

The numerical analysis of tailings flow follows the conservation law of mass and momentum. Since the heat change and transfer are negligible, the energy conservation law is not included. Furthermore, additional turbulence equations should be satisfied for the turbulent conditions caused by dam-break.

### 2.1. Mathematical Models

The mass conservation equation can be expressed in differential form as:

(1) $$\frac{\partial \rho }{\partial t}+div\left(\rho v\right)=0$$

(2) $$\rho =\rho_{s}c+\rho_{w}\left(1-c\right)$$

where *ρ* is the mixture density, $\rho_{s}$ is the solid density, $\rho_{w}$ is the liquid density, *c* is the mass concentration of tailings flow and *t* is the time. The operator *div* means the divergence, and ***v*** is the flow velocity vector. The tailings flow is assumed to be homogenous and incompressible, and its mixture density remains uniform and constant along the flow process. Therefore, Equation (1) can be simplified in rectangle coordinates as:

(3) $$\frac{\partial u}{\partial x}+\frac{\partial v}{\partial y}+\frac{\partial w}{\partial z}=0$$

Here *u*, *v* and *w* are velocity components in *x*, *y* and *z* directions.

Based on Newton’s second law, the momentum conservation equation, also known as Navier–Stokes equation, is derived in rectangle coordinates as:

(4) $$\{\begin{array}{c} \rho \frac{Du}{Dt}=\frac{\partial \sigma_{xx}}{\partial x}+\frac{\partial \tau_{yx}}{\partial y}+\frac{\partial \tau_{zx}}{\partial z}+F_{x}\\ \rho \frac{Dv}{Dt}=\frac{\partial \tau_{xy}}{\partial x}+\frac{\partial \sigma_{yy}}{\partial y}+\frac{\partial \tau_{zy}}{\partial z}+F_{y}\\ \rho \frac{Dw}{Dt}=\frac{\partial \tau_{xz}}{\partial x}+\frac{\partial \tau_{yz}}{\partial y}+\frac{\partial \sigma_{zz}}{\partial z}+F_{z} \end{array}$$

where the symbol $\frac{D}{Dt}$ is the substantial derivation and $\sigma_{xx}$, $\sigma_{yy}$ and $\sigma_{zz}$ are normal stresses in *x*, *y* and *z* directions. τ with different subscripts represents viscous stress components that depend on the rheological model. $F_{x}$, $F_{y}$ and $F_{z}$ are the body forces in *x*, *y* and *z* directions.

As the flow caused by dam-break is turbulent [30], the flow characteristics are random and usually portrayed by time average. An additional two-equation turbulent model, which includes the turbulent kinetic energy and the rate of turbulent dissipation transport equations [31], was adopted and written as:

(5) $$\{\begin{array}{c} \frac{\partial \left(\rho k\right)}{\partial t}+div\left(\rho \bar{u}k\right)=div\left[\left(\eta +\frac{\eta_{t}}{\sigma_{k}}\right)\cdot grad\;k\right]-\rho \varepsilon +\eta_{t}P_{G}\\ \frac{\partial \left(\rho k\right)}{\partial t}+div\left(\rho \bar{u}\varepsilon \right)=div\left[\left(\eta +\frac{\eta_{t}}{\sigma_{\varepsilon }}\right)\cdot grad\;\varepsilon \right]-\rho C_{2}\frac{\varepsilon^{2}}{k}+\eta_{t}C_{1}\frac{\varepsilon }{k}P_{G} \end{array}$$

where *k* is the turbulent kinetic energy, *η* is the dynamic viscosity, *ε* is the rate of turbulent dissipation transport and the operator *grad* means the gradient. The local dynamic viscosity $\eta_{t}$ and the turbulent kinetic energy generation term $P_{G}$ can be further expressed as:

(6) $$\{\begin{array}{c} \eta_{t}=\rho C_{\mu }\frac{k^{2}}{\varepsilon }\\ P_{G}=2\left[{\left(\frac{\partial u}{\partial x}\right)}^{2}+{\left(\frac{\partial v}{\partial y}\right)}^{2}+{\left(\frac{\partial w}{\partial z}\right)}^{2}\right]+{\left(\frac{\partial u}{\partial y}+\frac{\partial v}{\partial x}\right)}^{2}+{\left(\frac{\partial u}{\partial z}+\frac{\partial w}{\partial x}\right)}^{2}+{\left(\frac{\partial v}{\partial z}+\frac{\partial w}{\partial x}\right)}^{2} \end{array}$$

Here $\sigma_{k}$, $\sigma_{\varepsilon }$, $C_{1}$, $C_{2}$ and $C_{\mu }$ are adjustable constants with the value of 1.0, 1.3, 1.44, 1.92 and 0.09, respectively, in the simulation.

The Equations (1)–(6) can be rewritten in a general transport equation as the governing equation of the CFD method under the incompressibility condition. It is expressed as:

(7) $$\frac{\partial \phi }{\partial t}+\frac{\partial \left(u\phi \right)}{\partial x}+\frac{\partial \left(v\phi \right)}{\partial y}+\frac{\partial \left(w\phi \right)}{\partial z}=\frac{\partial }{\partial x}\left(\Gamma \frac{\partial \phi }{\partial x}\right)+\frac{\partial }{\partial y}\left(\Gamma \frac{\partial \phi }{\partial y}\right)+\frac{\partial }{\partial z}\left(\Gamma \frac{\partial \phi }{\partial z}\right)+S_{\phi }$$

where ϕ is the general variable, Γ is the diffusion coefficient and $S_{\phi }$ is the source term. Each Cartesian velocity component (*u*, *v*, *w*) satisfies its own transport equation but is non-linear and strongly coupled through the advective fluxes and pressure forces.

Equation (7) can be solved through the Finite Volume Method (FVM), which is the most commonly used method in fluid engineering [18,19]. The governing equations are discretised into algebraic expressions involving the physical quantities at the center of each control volume. The physical quantities are interpolated onto the faces with second-order accuracy. Preconditioning conjugate gradients (PCGs) and smooth linear algebraic solvers are used to solve the symmetric matrix and asymmetric matrix, respectively. The pressure-implicit with splitting of operators (PISO) algorithm was adopted to achieve the pressure-velocity coupling, which accelerates the convergence speed during the iterative process. More details about discretisation and coupled solve can be found in [32]. The initial and boundary conditions are specifically discussed in Section 3.2.

In addition, the roughness of the terrain surface in the ANSYS-CFX package is reflected by the roughness height. It depends on the geometry, size and arrangement of surface roughness elements. A technical roughness described by the equivalent sand-grain roughness is required as the input parameter.

### 2.2. Rheological Properties

Tailings slurry is a mixture of tailings and water. In the initial process of dam failure caused by overtopping, a mass of floodwater is discharged with tailings, so that the velocity of tailings is nearly equal to that of the water. Then a significant velocity discrepancy occurs between tailings and water, so that the tailings gradually deposit and separate from the flow. Consequently, the tailings flow becomes inhomogeneous, making it difficult to depict rheological properties. Thus, tailings flow is still treated as a homogeneous fluid throughout the whole evolution in many cases [33,34,35,36].

**Figure 1:**
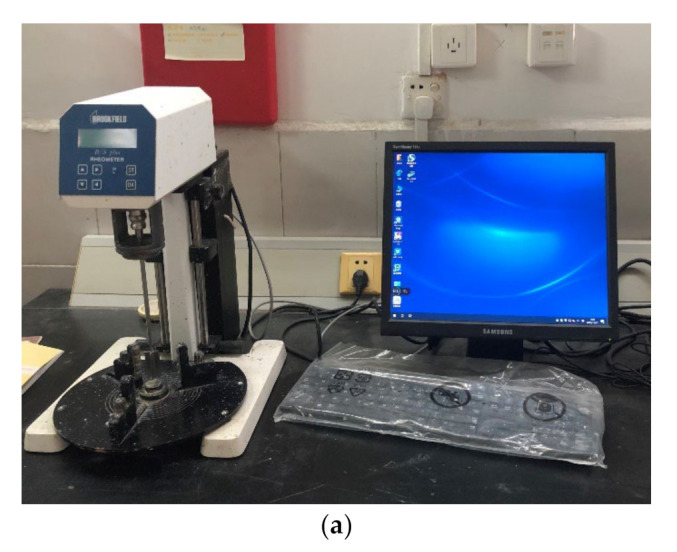

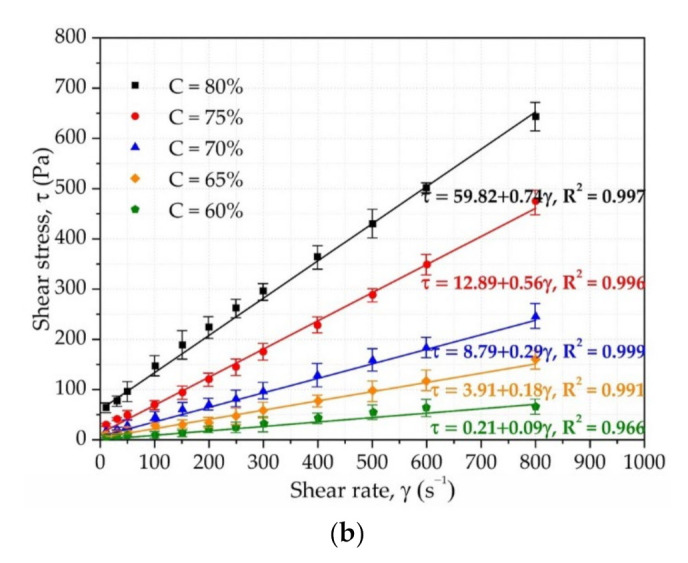
Testing apparatus and rheological results of slurry: (**a**) Rheometer testing apparatus; (**b**) Shear stress and shear rate relationships with fitted curves.

Homogeneous fluid mainly includes Newtonian fluid, Bingham fluid, expansible fluid, pseudoplastic fluid and yielding pseudoplastic fluid. Many researchers reported that Bingham fluid can represent tailings flow or debris in various situations [37,38,39,40]. For further discussion, the rheological properties of tailings slurry were measured through a rheometer in laboratory tests (Figure 1a). The specific gravity of the tailings (Gs) is 2.64, the average particle size (d_50_) is 0.11 mm and the fine particles (0.075~0.25 mm) accounted for almost two-thirds of the tailings. The tailings sample from the lead-zine mine tailings site was mixed with water to form the slurry for experiments. Five groups of slurry were prepared with the mass concentration (*C*) of 60%, 65%, 70%, 75% and 80%. The experimental data scatter points and fitting curves are exhibited in Figure 1b. It can be observed that the rheological results of slurry conform to the Bingham model, which can be described as follow:

(8) $$\;\tau =\tau_{0}+\mu \gamma$$

where τ is the viscous shear stress, $\tau_{0}$ is the yield shear stress, μ is the viscosity and γ is the shear rate. Yield shear stress and dynamic viscosity, the two rheology parameters of Bingham fluid, were the value of the intercept and slope of the line, respectively.

## 3. CFD Simulation

### 3.1. Validation

The flume test of a non-Newtonian dam-break flow conducted by Minussi [41] was used for the validation. The specimen of Carbopol solution (Lubrizol, Wickliffe, OH, USA), a Herschel–Bulkley fluid, was prepared and filled in the reservoir. A vertical gate was pulled upward by a hydraulic piston to cause the dam failure. Then the fluid stored in the reservoir was released and began flow. A guide grid was positioned on the lateral acrylic wall. A JVC digital camera (GY DV 500) was set to take a sequence of snapshots of the flow movement.

The CFD method was applied to simulate the above experiment for validation. A geometric model with the identical size of the flume was established and meshed with structure grids. The initial reservoir high was chosen to be 0.1 m. The Herschel–Bulkley model is given by Equation (9)

(9) $$\;\tau =\tau_{0}+k\gamma^{n}$$

where $\tau_{0}$ is the yield shear stress; *k* is the consistency, γ is the shear rate and *n* is the flow index. It is a generalised model of the Bingham model for *n* = 1.

The same rheological parameters from Minussi’s experiments were used ($\tau_{0}$ = 30.002 Pa, *k* = 4.297 Pa × *s^n^*, *n* = 0.479). The gate influence was disregarded and the flow began due to the pressure difference. The Volume of Fluid (VOF) method was adopted to analyze the multiphase flow. The turbulent terms were analyzed through a first-order upwind scheme. The transient terms were approximated by a second-order implicit scheme.

**Figure 2:**
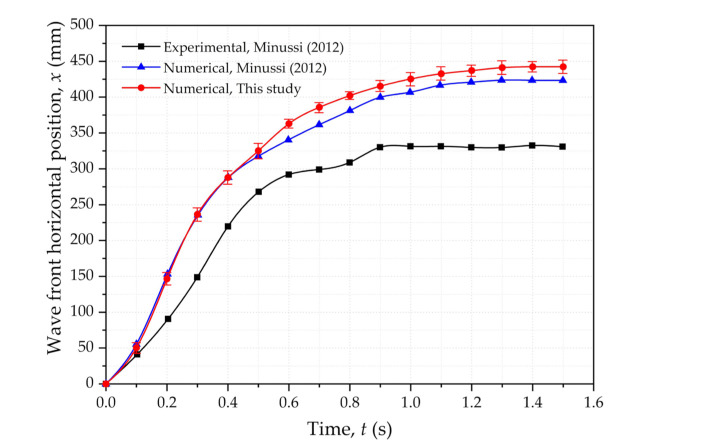
Numerical–experimental comparison of the wave front horizontal positions.

Figure 2 exhibits the numerical and experimental comparison of the wave front horizontal positions [41]. As shown in Figure 2, all the curves display the same trend of wave front horizontal positions rapidly increasing at the beginning and gradually levelling off. It is worth noting that numerical results presented greater distances than the experimental results. One possible reason is that the experimental data were captured at the sidewall, rather than at the flow’s centerline, leading to smaller values. In addition, lateral friction, gate influence and surface tension were disregarded in numerical simulations, contributing to the discrepancy. The numerical simulation in this study was consistent with experimental results for the use of complete motion equations of non-Newtonian fluid. Overall, the good agreement in this study between the calculated results and experimental results validates the applicability of the numerical method.

### 3.2. Dagangding Tailings Dam Failure

For exploring the variations in tailings flow characteristics against terrain features, a 3D simulation was carried out based on the topographic data of the Dagangding case.

**Figure 3:**
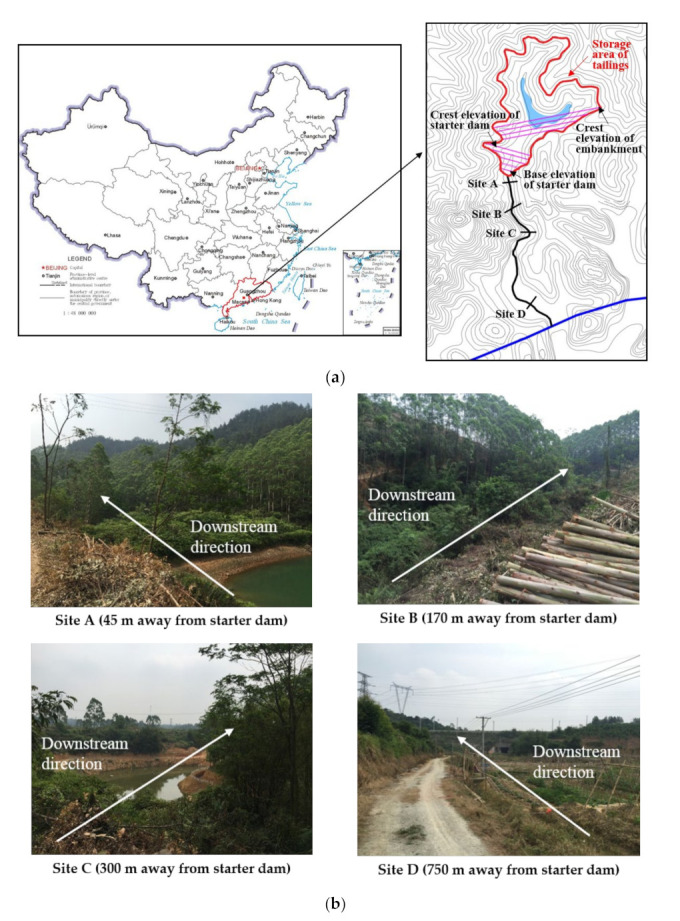
Location, topographic map, and downstream landforms of the Dagangding tailings dam: (**a**) Location and topographic map; (**b**) Landform of four typical sites.

The Dagangding tailings dam is located in Longkou Town, Heshan City, Guangdong Province, China, with 22°47′15″~22°50′23″N and 112°50′58″ to 112°53′28″E (Figure 3a). The region has a subtropical monsoon climate with an abundance of typhoons and rainstorms in summer and autumn. The average annual temperature is 22.6 °C, and the average precipitation is about 1700 mm.

The location and topographic map of the tailings dam can be seen in Figure 3a, and its characteristics are listed in Table 1. The tailings dam belongs to grade IV, and it was constructed by the upstream embankment method. Floods over a 200-year period were considered for flood control standards. The starter dam is a permeable rockfill dam about 17 m high, and the height of the embankment is 40 m. The Dagangding tailings dam has a total storage capacity of $358.86\times 10^{4}$ m^3^ and a catchment area of about 0.327 km^2^. The downstream gully meanders through different landforms (Figure 3b). Site A and Site B abound with shrubs and trees, and the landform between them is narrow and straight. The landform flattens gradually from Site B to Site C. Significantly, an apparent turning of about 80° appears at site C. Across site C for approximately 200 m, a wide and flat grassland with farmland around site D appears. A railway is situated 850 m away, and behind it is a village with about three hundred people.

**Figure 4:**
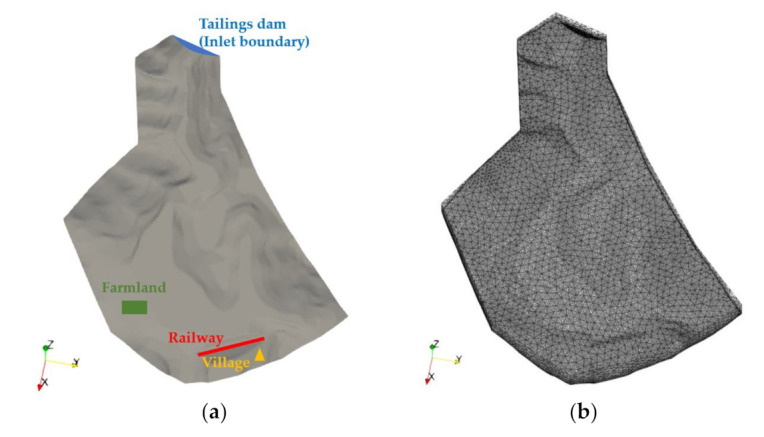
Diagram of the computational domain: (**a**) the geometric model; (**b**) the meshes.

The region from the dam breach to the railway was chosen as the calculation zone in the simulation. The erosion influence was out of scope in this paper. For characterising the actual terrain, the ground surface was interpolated from contour data of the topographic map through MIDAS-GTS software (version of 2013 R1). Then the geometric model was conducted and meshed in the ICEM software (version of 14.5) (Figure 4). Tetrahedral meshes were used in the majority area, with a maximum volume mesh size of 30 m^3^ and a maximum face mesh size of 10 m^2^. The pentahedral prism meshes were adopted near the boundary of the inlet and ground surface, with a maximum volume mesh size of 10 m^3^ and a minimum volume mesh size of 2 m^3^. The mesh consisted of 543,243 elements and its total number of nodes was 109,680. The Bingham model was considered for the case based on rheological results. The rheological values at a mass concentration of 70% were selected for a conservative estimation. Parameters in the numerical analysis are displayed in Table 2.

Since the process of breach development involved considerable uncertainty, it was assumed that the dam collapsed instantaneously, ignoring the dynamic hydrograph associated with the breach development. That is, the geometry of the breach (length, width and height) after dam-break was assumed to be constant.

Rather than the entire volume of the reservoir dam being discharged, some tailings remained in the tailings pond after dam failure. The quantitative assessment of potential consequences caused by tailings flow requires an appropriate estimation of the discharge volume of tailings slurry. A common conservative approach is to choose the deepest dam break section, which collapses along the residual friction angle of the starter dam. After calculating the deepest dam break section, it was concluded that the discharge volume of the tailings slurry was one third of the total storage capacity, with a value of $1.20\times 10^{6}$ m^3^. Then the breach width was calculated by the empirical equation [42] as below:

(10) $$b=K{\left(W^{\frac{1}{2}}B^{\frac{1}{2}}H\right)}^{\frac{1}{2}}$$

where *W* is the discharge volume and equals $1.20\times 10^{6}$ m^3^; *K* is the empirical coefficient for the materials of starter dam and equals 0.65; *B* is the crest width and equals 497.7 m; *H* is the head of the dam-break and equals 57.0 m; calculating the breach width *b* equals 76.7 m.

The maximum discharge rate at breach (*Q_m_*) has an important influence on the final inundation extent. On the basis of dam-break flood theory and debris flow correction [43], *Q_m_* was calculated by:

(11) $$\{\begin{array}{c} Q_{m}=\left(1+\emptyset \right)D_{m}Q_{w}\\ \emptyset =\frac{\rho -1}{G_{s}-\rho }\\ Q_{w}=0.27{\left(\frac{L}{B}\right)}^{\frac{1}{10}}{\left(\frac{B}{b}\right)}^{\frac{1}{3}}\sqrt{g}b{\left(K^{\prime }h\right)}^{\frac{3}{2}}\\ K^{\prime }=1.4{\left(\frac{bhH_{0}}{B}\right)}^{\frac{1}{3}} \end{array}$$

where ∅ is the correction factor for tailings weight; ρ is the fluid density and equals 1.82 g/cm^3^; $G_{s}$ is the specific gravity of tailings and equals 2.64; $D_{m}$ is the empirical coefficient for clogging of the drainage system and equals 1.5; *L* is the crest length and equals 375 m; *K*’ is the empirical coefficient for dam geometry; *h* is the average height of residual after break, $H_{0}$ is water depth before the dam break and equals 5 m; calculating the maximum discharged rate of water at dam breach *Q_w_* is 1312.9 m^3^/s and then the maximum discharge rate at breach *Q_m_* equals 3892.9 m^3^/s.

According to the relationship between the instantaneous dam-break flow process, the maximum discharge rate at breach and the discharge volume, the flow process curve at breach is approximately simplified as a quaternary parabola [44] as below:

(12) $$Q=Q_{m}{\left(\frac{Q_{m}}{5W}t-1\right)}^{4}$$

where *Q* is the discharge flow rate of tailings slurry at dam breach at the time *t.*

**Figure 5:**
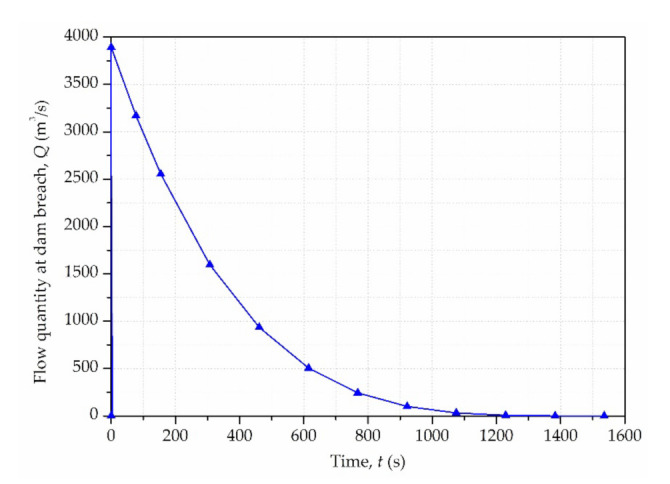
The hydrograph of tailings flow at dam breach.

In this case, the hydrograph of tailings flow at dam breach is shown in Figure 5. The inlet boundary condition was set as bulk mass flow rate, obtained from the product of the discharge flow rate (*Q*) multiplied by the fluid density (ρ).

The outlet and top surface were free boundaries. According to Davenport’s classification of major shrub cover, the ground was set with a roughness height of 0.03 m. Surrounding surfaces used smooth wall boundaries.

It should be noted that different assumptions about the material model and boundary conditions result in distinct numerical results on runout distance, inundation area, etc. In this study, the tailings flow characteristics linking practical topographic features were investigated in a conservative scenario combining the simplified model, constant parameters and supernumerary influx.

## 4. Results and Discussion

### 4.1. Flow Velocity and Runout Distance

**Figure 6:**
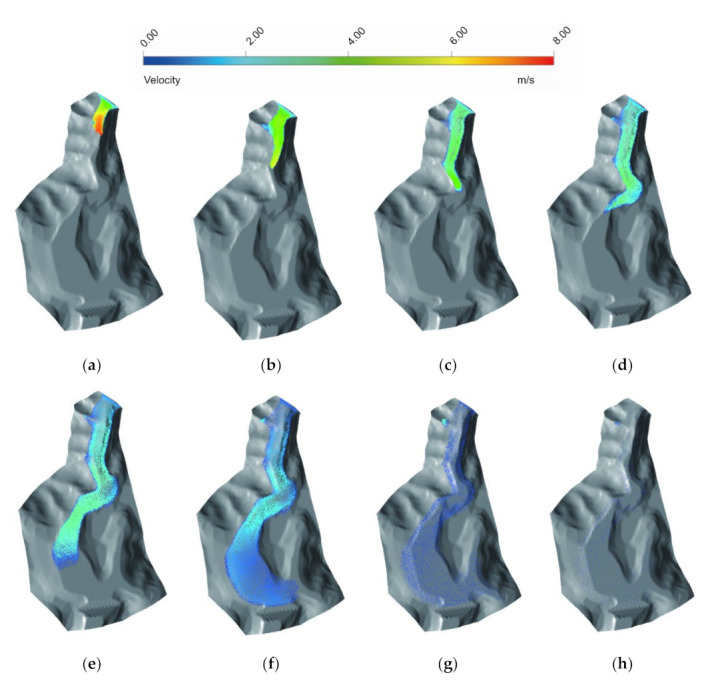
Velocity variations at different times for the process of the Dagangding tailings flow: (**a**) *t* = 10 s; (**b**) *t* = 20 s; (**c**) *t* = 40 s; (**d**) *t* = 80 s; (**e**) *t* = 160 s; (**f**) *t* = 320 s; (**g**) *t* = 640 s; (**h**) *t* = 1280 s.

Flow velocity is an important index to evaluate the characteristics of tailings flow. As shown in Figure 6, the velocities of tailings flow decreased along the runout path. In the initial 10 s, the flow velocity increased rapidly, and the peak value of about 8 m/s was identified at the flow front (Figure 6a). After the peak velocity, it slowed down gradually due to energy dissipation from the friction and collision during the propagation (Figure 6b). Soon after, the difference between terrain and altitude enabled a velocity drop in the tailings flow (Figure 6c). Then a rapid decline in flow velocity was noted with a sharp turning (Figure 6d). After the deflection, the tailings flow underwent a decelerated process to 2.65 m/s (Figure 6e). At 320 s, the flow spread over the flat area where the farmland was located. At this stage, the front flow was approaching but had not crossed the railway (Figure 6f). Thereafter, the flow began to stabilise, moving slowly downstream, and divided into two branches (Figure 6g). Finally, the tailings flow stopped at *t* = 1280 s (about 22 min), with a runout distance of approximately 750 m (Figure 6h). It can be inferred that the village behind the railway would not be affected, as the volume and velocity of the tailings flow had reduced significantly.

**Figure 7:**
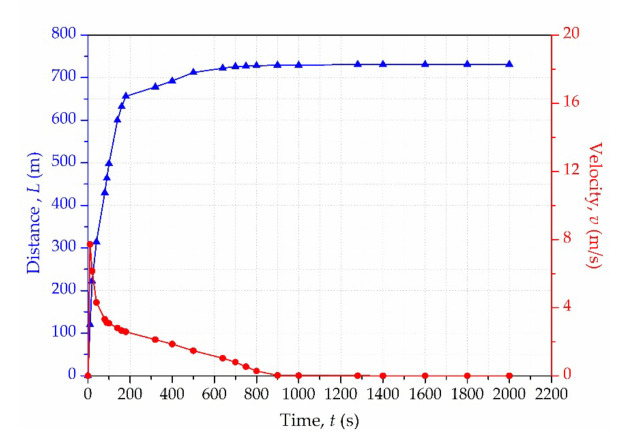
Distance and velocity of the front flow during the evolution process.

To further investigate the characteristics of tailings flow, Figure 7 exhibits the time history curves of the flow distance and velocity. The blue curve represents the runout distance of the front flow, and the red curve indicates the velocity of the front flow. It took approximately 200 s for the front flow to reach most distances. Afterwards, the movement gradually stopped over 600 s. The runout distance was approximately 750 m. It can be noted that the velocity of front flow reached a peak velocity of about 8 m/s, and decreased rapidly to about 3 m/s during the initial 100 s. Then the velocity gradually dropped in the following 700 s, which conformed to the effect of flatter terrain. Eventually, the flow stopped at about 1280 s. In addition, it was observed that the time response of distance lagged behind that of velocity.

### 4.2. Inundation Area and Depth

**Figure 8:**
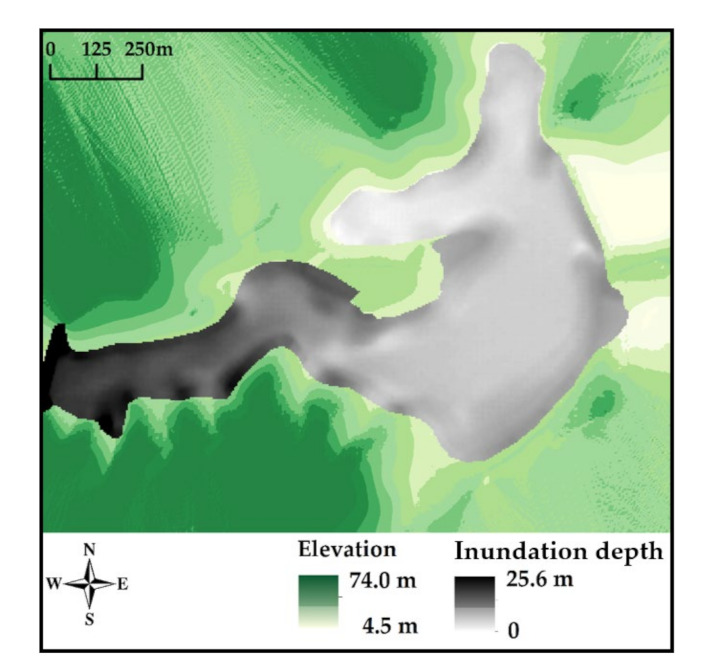
Inundation area and thickness for the process of the Dagangding tailings flow.

The final inundation area and depth for the Dagangding tailings flow are presented in Figure 8. The elevation is shown in a colour band from light to dark green. The inundation area is represented by the grey colour. Furthermore, the inundation depth along the runout path is depicted in different shades of grey. It can be seen that most tailings were deposited near the dam breach, due to the gravity effect. The maximum inundation depth was 25.6 m. The inundation depth was greatly influenced by the variations in the downstream terrain. Tailings accumulated at sharp corners and spread out in flat areas, such as the farmland.

**Figure 9:**
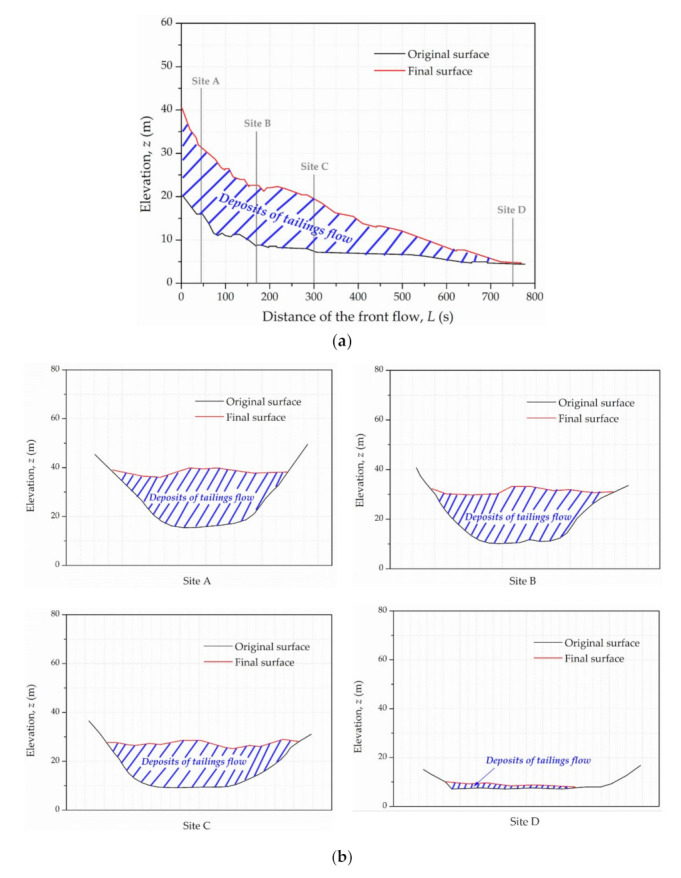
Numerical results of the Dagangding tailing flow: (**a**) final profile; (**b**) cross sections of four typical sites.

In addition, the final profile of the Dagangding tailings flow along the flow path is portrayed in Figure 9a. The black curve means the elevation of the original surface, and the red curve refers to that of the final surface. Furthermore, their difference represents the deposit of the tailings flow. This proves the decreasing trend of the inundation depth along the path. The deposition depends on both energy dissipation and the variation of terrain. The cross sections of four typical sites, associated with Site A, Site B, Site C and Site D in Figure 3, are shown in Figure 9b. Site A and Site B were located in the valley with an obvious elevation difference from the surrounding hills. Site C displayed the sharp turning of the flow path. Site D was relatively wide and flat near the farmland and had some power facilities. The average inundation depth remained relatively stable between Site A (15.8 m), Site B (14.3 m) and Site C (13.6 m), but reduced swiftly at Site D (0.8 m). When the front flow reached the furthest distance of about 750 m, the inundation depth was about 0.3 m. Since the railway was approximately 2 m above the original surface elevation, the village behind it would not be affected.

## 5. Protection and Control Measures

**Figure 10:**
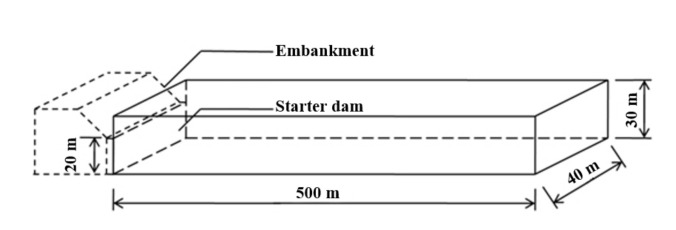
Simplified model of a tailings dam and the downstream channel.

Tailings flow significantly impacts people’s livelihood and the surrounding ecological environment. For this reason, engineers have proposed several control measures, such as setting a check dam and planting trees or grasses. To investigate the effects of the two measures, a simplified numerical model (Figure 10) was established. The starter dam and embankment heights were 20 m and 10 m, respectively. A regular rectangular channel was set measuring 500 m in length and 40 m in width. The rheological parameters were obtained from previous experiments at a mass concentration of 70%. The inlet mass flow rate at dam breach was calculated by Equation (9) with a released volume of $W=5\times 10^{4}$ m^3^.

### 5.1. Setting the Check Dam

Setting the check dam is essential for resisting the dynamic force of dam-break-type flows [45,46,47,48]. A gravity impermeable check dam with a trapezoid cross section was considered in numerical simulation. In order to evaluate its blocking effect with various locations and heights, six cases are summarised in Table 3. Case 1 (with no check dam) was used as the base case. Cases 2–6 were set at different locations (100 m, 200 m, 300 m) with various heights (2 m, 3 m, 4 m).

**Figure 11:**
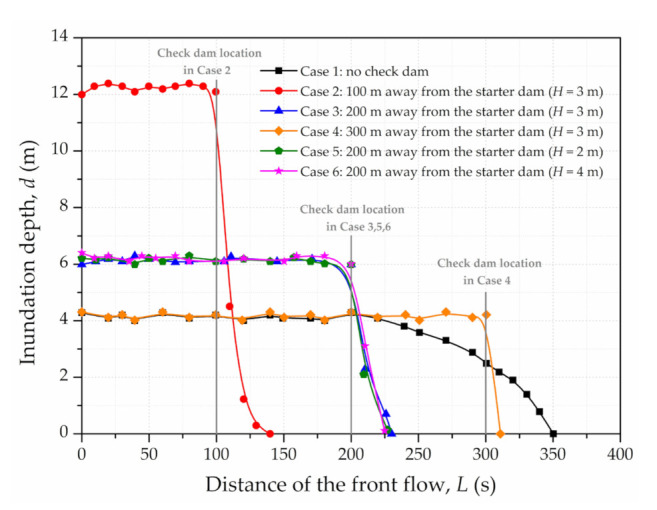
The inundation depth along the downstream channel.

As shown in Figure 11, the front flow distances shortened remarkably compared with the base case (Case 1). Additionally, the distance increased with increasing check dam locations. It is evident that distance reductions of 61% (Case 2), 35% (Case 3) and 13% (Case 4) occurred relative to the base case. However, the fluid level was raised before the check dam and then dropped quickly behind it. Furthermore, the maximum inundated thickness decreased with increasing check dam locations, which are 12.0 m, 6.2 m and 4.1 m of Case 2, Case 3 and Case 4, which are 12.0 m, 6.2 m and 4.1 m of Case 2, Case 3 and Case 4. Therefore, a suitable location is essential, neither too far away nor too close to the dam breach. In addition, it is noteworthy that similar curves appear for the check dam at the same location with different heights. Both the front flow distance and the inundation depth of three cases (Case 3, 5 and 6) presented quite closed values. Thus, the height was relatively unimportant when setting the check dam. It is concluded that a check dam can effectively intercept the tailings flow with proper design, consistent with the results of existing studies [28,48]. Unfortunately, a scenario involving multiple check dams is beyond the scope of this article.

### 5.2. Planting Trees or Grasses

Planting trees or grasses is a generally accepted measure for protecting against the tailings flow hazard [49,50]. Different types of plant cover have different surface roughnesses. The roughness length (*Z***_0_**) is used to describe the surface roughness in numerical simulation. The roughness length (*Z***_0_**), an aerodynamic term defined numerically as the height where the wind velocity is zero (it does not exist physically), reveals the roughness characteristics of the terrain [51]. It is generally independent of airflow and only depends on the geometry, size and arrangement of surface roughness elements. It is difficult to obtain real values of the surface roughness under heterogeneous conditions, therefore, practical estimation is often based on published values for roughness of similar terrain elsewhere [52]. Since Davenport’s classification of effective terrain roughness has proven to be reliable [51,52], four typical classes (Class 3, Class 4, Class 6 and Class 7) in the classification were selected to represent four types of plant cover (Type 1, Type 2, Type 3 and Type 4), as shown in Table 4.

**Figure 12:**
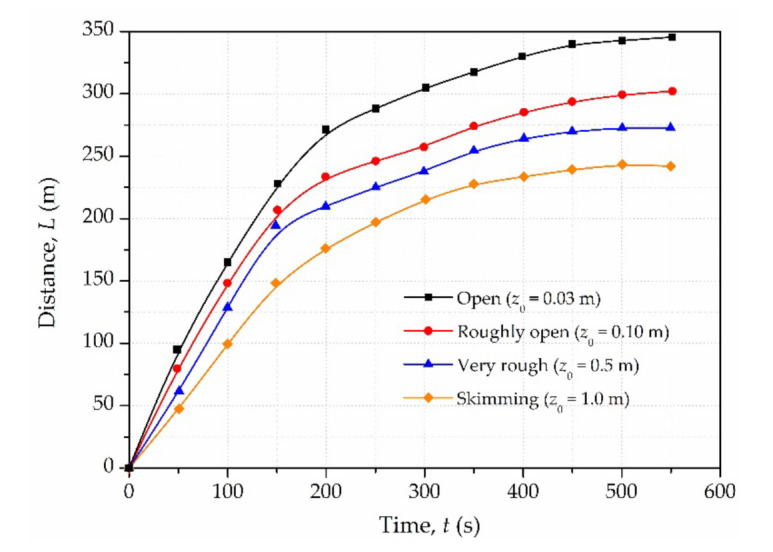
Distance–time history curves of different types of plant cover.

Figure 12 shows the distance–time history curves of different types of plant cover. It is evident that the variation trend is similar. The front flow distance increases rapidly in the initial 200 s, and a gradual decline in velocity is noted with the slow-growing distance. Planting trees or grasses can shorten the runout distance while protecting the ecological environment. Moreover, the runout distance reduces with an increase in the roughness length. Taking Type 1 (grass cover) and Type 4 (tree cover) for comparison, it can be seen that the runout distance of Type 4 reduces by 32.02% compared with that of Type 1. Therefore, the type of plant cover plays an important role in mitigating the impact of tailings flow. In addition, the specific implementation should be carried out according to local conditions, such as terrain, slope, soil thickness and others.

## 6. Conclusions

Tailings flow from dam breaks represent a complex problem of physical mechanics and fluid properties. In this paper, tailings flow is assumed to be an incompressible non-Newtonian fluid following the Navier–Stokes governing equations. The rheological properties of tailings slurry with different mass concentrations were studied by laboratory experiments. Subsequently, the results were further used in a numerical simulation. The method was validated with flume experiments of a non-Newtonian dam-break flow with good consistency. A framework combining the three-dimensional CFD model with complex terrain reconstruction was established to dig into the mechanism of the tailings flow. Finally, protection and control measures for potential tailings flow hazards were discussed. The following conclusions can be drawn:

(1) The experimental results signify that this tailings slurry, composed of lead-zine mine tailings with about two-thirds fine particles (0.075~0.25 mm), conformed to the Bingham model. The two rheological parameters of the Bingham model, yield shear stress and dynamic viscosity, were obtained. A non-linear relationship between the mass concentration and yield shear stress or dynamic viscosity was observed.
(2) The variation in flow characteristics (flow velocity, runout distance, inundation area and depth) against the terrain features in the Dagangding case were analyzed within our framework. Results show that a sharp turning can bring in a rapid decline in flow velocity, implying a flow regime transition. Varying roughness can make a difference when referring to the runout distance. The runout distance and the roughness form a negative relationship. A similar relationship can be also seen between inundation depth and sudden flattening. A decrease in sudden flattening contributes to higher aggregation of the tailings. Meanwhile, the flow lasted about 1280 s (about 22 min), possessing a maximal runout distance of about 750 m, which shows that the downstream railway and concerned villages would not be affected in a conservative scenario.
(3) The effects of protective measures, including setting a check dam and planting trees or grasses on the tailings flow, were studied. The development of the tailings flow can be effectively constrained by a properly designed check dam. Its location, rather than its height, plays a dominant role in blocking the flow. In addition, planting trees or grasses can shorten the runout distance while protecting the ecological environment. in this study, the types of plant cover influenced the runout distance of the tailings flow, and the distance of Type 4 (tree cover) was less than (reduced by 32.02%) that of Type 1 (grass cover).

Even though the proposed numerical framework is beneficial for investigating the tailings flow, there also remain some limitations. In this research, the material model was assumed to be isotropic and incompressible, and the mechanical behaviour conformed to an ideal Bingham model. Some state variables (fluid density and mass concentration) were time-independent and may have introduced computational errors. The authors intend to add the optimisation of the material model to their future work. Moreover, the boundary condition can be seen as another aspect of the methodology to be improved. The current iteration scheme experienced difficulty in mimicking the influx for the inlet boundary condition. It seemed to be unable to accurately characterise the breach development, discharge volume and discharge flow rate along with time up to date. For this reason, the authors made some assumptions to compensate, for example by calculating the discharge volume from the deepest dam break section. Such a conservative condition may overestimate the total flux. From a future perspective, the authors plan to introduce more failure criteria for a concise discharging volume calculation. The final point concerns the roughness setting of the ground. Uniform roughness was assumed herein, but a grid-wise distribution of the roughness can be adopted in the future. In summary, the authors believe the major factors for further improvement are the variation in material properties, the dam breach process and the refinement of the ground condition.

## Figures and Tables

**Table 1:** Characteristics of the Dagangding tailings dam.

Concept	Contents or Values
Grade	IV
Construct method	Upstream embankment method
Flood control standard	Flood in a 200-year
Height of starter dam	17 m
Height of embankment	40 m
Whole storage capacity	$358.86\times 10^{4}$m^3^
Catchment area	0.327 km^2^

**Table 2:** Parameters in numerical analysis.

Parameters	Values
Fluid density, *ρ* (g/cm^3^)	1.82
Mass concentration, *C* (%)	70
Yield stress, τ_0_ (Pa)	8.79
Viscosity, μ (Pa·s)	0.29

**Table 3:** Summaries of setting check dam in numerical simulation.

Name	Check Dam Location ^1^, *L* (m)	Height of the Check Dam, *H* (m)
Case 1 ^2^	0	0
Case 2	100	3
Case 3	200	3
Case 4	300	3
Case 5	200	2
Case 6	200	4

^1^ Location refers to the distance away from the starter dam; ^2^ Case 1 refers to the condition of no check dam.

**Table 4:** Summaries of roughness length.

Classes	Roughness Length, *Z*_0_ (m)	Types of Plant Cover
**Class** 1. Sea	0.0002	\
**Class** 2. Smooth	0.005	\
**Class** 3. Open	0.03	Type 1. Grasses cover
**Class** 4. Roughly open	0.10	Type 2. Low crops cover
**Class** 5. Rough	0.25	\
**Class** 6. Very rough	0.5	Type 3. Shrubs cover
**Class** 7. Skimming	1.0	Type 4. Trees cover
**Class** 8. Chaotic	≥2.0	\

## Data Availability

Data is contained within the article.
